# Human rotavirus in Iran; molecular epidemiology, genetic diversity and recent updates on vaccine advances 

**Published:** 2019

**Authors:** Shadi Tavakoli Nick, Seyed Reza Mohebbi, Amir Ghaemi, Seyed Masoud Hosseini

**Affiliations:** 1 *Foodborne and Waterborne Diseases Research Center, Research Institute for Gastroenterology and Liver Diseases, Shahid Beheshti University of Medical Sciences, Tehran, Iran*; 2 *Department of Microbiology and microbial biotechnology, Faculty of life Sciences and biotechnology, Shahid Beheshti University, Tehran, Iran *; 3 *Gastroenterology and Liver Diseases Research Center, Research Institute for Gastroenterology and Liver Diseases, Shahid Beheshti University of Medical Sciences, Tehran, Iran*; 4 *Department of Virology, Pasteur Institute of Iran, Tehran, Iran*

**Keywords:** Rotavirus, Gastroenteritis, Diarrhea, Vaccine, Immunization program, Molecular epidemiology

## Abstract

Human rotavirus is the predominant pathogen causing gastroenteritis in infants and children younger than 5 years of age globally. Before introduction and implementation of rotavirus vaccine, more than frothy percent of all caused acute gastroenteritis hospitalization and nearly half a million deaths per year was occurred due to Rotavirus infection mostly in the low-income countries. Rotaviruses are divided in G and P genotypes, based on two genomic segments’ nucleotide sequences VP7 and VP4, respectively. Currently, 27 G and 37 P types have been described; among them G1 to G4 and G9 and P[8], P[4], and P[6] genotypes are the most prevalent circulating rotavirus strains globally. Molecular epidemiological surveys revealed that G1P[8] is the predominant genotype in Iran, although other genotypes have also been reported. Rotavirus vaccine was recommended by the World Health Organization as a necessary part of national childhood immunization programs in 2009. Rotarix (monovalent) and RotaTeq (pantavalent) are two oral vaccines that have been available in more than one hundred countries around the world to control the viral infection and reduce the cases of diarrheal diseases. This article provides a review of frequency, molecular epidemiology and current situation of Rotavirus genetic diversity Iran. In addition, recent advances in rotavirus vaccine research are discussed.

## Introduction

 Acute gastroenteritis (AGE) is one of the most important reasons of death in pediatric populations worldwide. Every year AGE causes two million deaths in the group of infants and young children around the world. An estimated 800 000 children under 5 die from diarrhea annually. Viruses are extremely significant causes of AGE in young children especially under 5 years of age. Group A rotavirus, enteric adenoviruses, norovirus, sapovirus and human astrovirus are established causes of AGE (1-3).

The World Health Organization (WHO) attributed a globally estimate of 17% mortality because of diarrhea in infant and the young children (less than five years old) with 40% in Africa only. Diarrheal diseases are a significant cause for morbidity over different age groups. Rotavirus (RV) is accountable for most of acute gastroenteritis.

Annually 114 million diarrhea diseases, 24 million clinic attendance, 2.4 million hospitalizations and 453,000 deaths are reported around the world. This high rotavirus-associated mortality occurs generally in South Asia and Africa. Mortality is unusual in developed countries, however when it comes to society impact diarrhea has a noticeable effect in terms of healthcare and medical costs (4-8).

In developing regions, because of insufficient domestic sanitation and areas with inexistent access to the healthcare infrastructure, the average age of early exposure to RV is around 6 to 9 months old. In the developed world, where public health standards are considerably satisfactory, the average age of RV primary infection may occasionally be delayed until the age of 2–5 years. Acute gastroenteritis is an extremely common disease which often starts with fever, nausea and vomiting and followed by severe abdominal pain and cramps and frequent watery diarrhea. Checking the patient’s history is important for diagnosis of AGE. 

When diarrhea occurs with vomiting, it may induce serious condition of dehydration. The best clinical indicators of dehydration include thirst, becoming irritable, weakness and fatigue, lethargy, dry mouth and tongue and also dry skins. Dehydration is an important complication of rotavirus, and this situation can lower blood volume, collapse and finally death (9-11).

**Viral Structure**

Rotaviruses are a genus of double-stranded RNA viruses of *Reoviridae* family. The viral architecture is consisted of a non-enveloped icosahedral capsid as a triple-layered particle (TLP). By electron microscopy analysis, TLPs look similar to a wheel, and this appearance is the main reason for the title “Rotavirus” (12, 13).

The capsid firmly wraps viral genome that is comprised of 11 segments of double-stranded RNA. The segments encode 6 structural viral products (VP1 to VP4, VP6 and VP7) and 5 or 6 non-structural proteins (NSP1 to NSP6) (9, 14-17). 

Each double-stranded RNA segment encodes a single structural (VP1 to VP7) or nonstructural proteins (NSP1 to NSP4) other than segment 11, that encodes for NSP5 and NSP6 (1). The structural proteins can constitute rotavirus virion. The nonstructural proteins, are only involved in cells infected by rotavirus (14) . The inner and intermediate layers of the capsid are composed consequently of VP2 and VP6 structural protein, the outer part of the capsid is composed of VP4 and VP7 structural proteins which consist of neutralizing antigens that are classified into G and P serotypes, respectively via neutralization tests. In addition to these serotypes, genotypes G and P, based on the different genes of VP7 and VP4 have been defined to at least 27 G-types and 37 P-types. Recently, in most location around the word including Iran, G1P[8], G2P[4], G4P[8], G3P[8], and G9P[8] are common source of about 90% of all human infections associated with rotavirus , prevalence of G1P[8] is the highest among the other genotypes (8, 9, 15, 18, 19). 

During the first occurrence of rotavirus infection, rotaviruses are shed continuously for several days in high concentrations (>10¹² particles/gram) in patients’ stool and vomit. Transmission occurs mainly by the fecal-oral route in direct manner from person to person, or in indirect manner through contaminated fomites (19).

**Laboratory diagnosis**

Diagnosis of etiological rotavirus gastroenteritis needs laboratory confirmation. Different diagnostic assays are commercially accessible: enzyme immunoassays for detecting antigens of rotavirus in stool specimens in direct manner are used generally, despite of the less sensitive, but rapid and easy-to-use test strips and latex agglutination method. Reverse transcription polymerase chain reaction (RT-PCR), that is very sensitive in diagnosis low concentrations of rotavirus in stool specimens, is also used for identification and further differentiation (19, 20).

**Figure 1:**
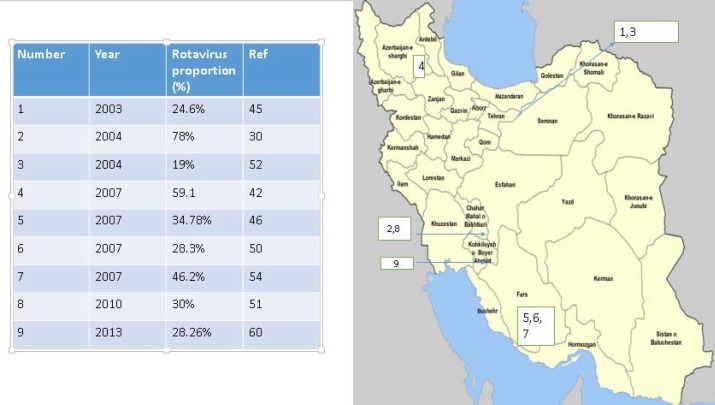
Left) Prevalence of Rotavirus infection in different studies from various parts of Iran. Right) Illustration of the province and location of previous surveys. Each number on the Iran’s map shows the relevant references from the left table

**Figure 2:**
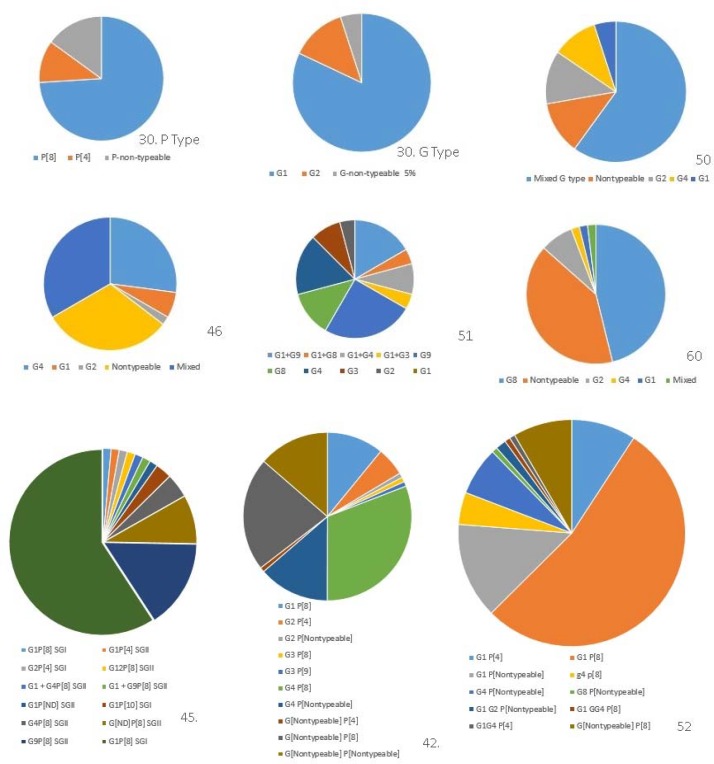
G type and P type distributions of Rotavirus isolates in previous studies in Iran. The number under each pie chart shows the reference and different colors illustrate G and P types founded in previous studies

**Epidemiology**

Many studies performed to investigate rotavirus epidemiology in the world. Several researchers determined and reported the rates of rotavirus related infections in Latin America (30%), Europe (40%), and Africa and Middle East (34%–40%) (21-24). WHO estimated the rate of rotavirus related gastroenteritis approximately between 14% to 45% in Iran, Iraq, Syria, Egypt, Oman, Jordan, Yemen, Libya, Morocco, and Tunisia (25). 

Latipov *et al. *in 2011 reported rotavirus positivity in Kazakhstan around 15%, in Kyrgyzstan about 36% and Uzbekistan almost 49% (26).

Rotavirus infection rate reported 20%–30% in some Asian locations including India, Hong Kong, South Korea and Bangladesh, but in China, Vietnam, Japan, Taiwan, Myanmar, and Thailand the rate of Rotavirus infection was 43%–58%. Rahman *et al.* in 2007 reported the rate of rotavirus related infection in Dhaka and Matlab (cities in Bangladesh) around 25.2% and 23.3% respectively, and (24.4%) samples had positive results for group A rotavirus VP6 antigen (27).

Malek *et al*. in 2010 reported the rate of rotavirus infection among patients (children) with acute gastroenteritis in Eastern Mediterranean around 40% for inpatients and about 23% for outpatients. This research revealed highest rate of rotavirus gastroenteritis in Syria (61%) and lowest rate in Tunisia, Egypt and Saudi Arabia (16%–23%) (28). Amini *et al.* studied 915 children less than five years in Tehtan during March 1986- August 1987. Rotavirus related infection was found in %25 of patients and %28 of rotavirus infections were occurred in 7-24 months old. The most rate of rotavirus infection (%30-%37) was occurred between April and May and the lowest (%7) was occurred during December and January (29).

Khalili *et al.* in 2004 studied children under 5 years old with acute diarrhoea in Shahrekord, Iran and rotavirus was reported in 78%. In this recent study 95% of rotaviruses were determined as G types and 85% as P type. The frequency of P type was P(8) (74%), P(4) (11%), (15%) were P-non-typeable respectively, and the frequency of G type was G1 82%, G2 13%, and 5% were G-non-typeable (30). Saeb *et al.* in 1997 determined 35.5% rotavirus in children under 5 years old with acute diarrhoea in Zahedan (31). Habibi *et al. *in 2004 studied 180 Fecal sample of newborns and young children with acute gastroenteritis of two hospitals in Tehran, 36.7% rotavirus Type A was reported (32). Moradi *et al*. conducted an epidemiological and virological survey in young children under 6 years old in 1993 of Ali-Asghar hospital (Zahedan- Iran) rotavirus was detected in 29.2% of patients. The highest rate of rotavirus related infection was between August (40% ) and September (39.5%) and the lowest in December (11.1%) (33).

Modares et* al. * reported the rate of rotavirus group A infection in hospitalized children < 5 years old in Tehran during January 2002 to April 2004 28.4%, and 70% of rotavirus infections were in children< 2 years old with the highest rate of infection in 6 to 12 months. The most of Rotavirus infections were occurred in February, January, December ( cool seasons) (34). 

Kordidarian *et al.* in 2007 studied the incidence of rotavirus associated nosocomial infection in children around 3 to 24 months old during November to April 2003 and 2004 in one of the Isfahan’s hospitals in Iran. The incidence of rotavirus associated nosocomial infection was 26.25%, and 15% of studied children which rotavirus antigen was diagnosed in their stools had acute diarrhoea (symptomatic nosocomial infection), and 11.25% of studied children had asymptomatic infection (35).

Kazemi *et al.* conducted a study in children (up to 5 years) with acute diarrhea hospitalized in Isfahan from December to November 2003 and 2004. In this study rotavirus was detected 30.8% children of the study population with acute diarrhea 84.2% of cases with rotavirus related diarrhea were under 2 years old with the highest incidence in children 6-12 months old. The highest rate of rotavirus related infection (41.4%) was in winter. Rotavirus associated gastroenteritis occurred with the highest rate in Bottle-fed children and children who were receiving care at child care centers (36).

Zarnani *et al.* in 2002 performed a study to determine the role of rotavirus in children younger than 5 years suffering from acute gastroenteritis in two children medical center and one general hospital in Tehran. In 15.3% of children with acute diarrhea rotavirus antigen was diagnosed with highest incidence in children between 6-12 months of age. Rotavirus infection occurred with lower frequency in breast fed infants than bottle fed. The rate of detection was higher in the spring than summer, although the rate of hospitalization was the highest in winter (37).

Samarbafzadeh *et al.* conducted a study in Ahwaz (city of Iran) from November 2001 to March 2002 in inpatient and outpatient children between 1 to 24 months old. Rotavirus was reported 26.3% of outpatients and 36.5% of hospitalized patients. In this study population, the frequency of rotavirus was 29.5% with highest incidence in children between 7 to 12 months old that showed a potential link between age and rate of rotavirus infection (38).

Kazemi *et al.* reported the incidence of rotavirus with acute diarrhea 32% in Zanjan and the highest prevalence were between children of 6-24 months of age (39).

Hamkar *et al.* reported the incidence of rotavirus with acute diarrhea 62% in Mazandaran (40).

Taremi *et al. *conducted an epidemiological and virological survey in young children under 5 years old in 2005 with acute diarrhea admitted in Markaz Tebbi pediatric hospital (Tehran-Iran). The incidence of rotavirus was 25.3S% of study population with highest incidence in children less than 2 years of age. The highest rate of rotavirus related infection was during winter (46.2%) (41). 

Eesteghamati *et al.* conducted a hospital-based survey of acute diarrhea at 5 hospitals in different regions of Iran (Tabriz and Mashhad in the northern region of Iran, Tehran in the central region of Iran, and Shiraz and Bandar Abbas in the southern region of Iran) From 1 May 2006 through 30 April 2007 in children less than 5 years of age who were hospitalized overnight for diarrhea. Of 2198 children enrolled to the hospital for diarrhea, 59.1% was positive for rotavirus totally with the highest prevalence (85%) in children less than 2 years of age, with the peak incidence of acute rotavirus related infection from September to January. The prevalence of rotavirus was 44% in Tehran, 49% in Tabriz and 49% in Mashhad (subtotal northern region of Iran: 49.2%) 66.3% in Shiraz and 63.3% in Bandarabbas (subtotal southern region of Iran: 65%). 110 positive samples for rotavirus were genotyped, among the positive samples. The most frequently rotavirus genotype detected was G4P[8] (30.9% of strains). P[8] with G nontypeable 21.8%, G4 with P nontypeable 13.6%, G1[P8] 10.9% and G2P[4] 5.5% were detected respectively (42) .

Savadkoohi *et al.* in 2007 determined the prevalence of viral gastroenteritis in Babol, Iran. They reported the prevalence of rotavirus in this recent study was 61.1% with the highest incidence (29.8%) in children between 6 and 12 months of age. Rortavirus infection with the rate of 53.5% was occurred in urban region and 46.5% in rural region. They showed that the prevalence of rotavirus infection in the summer was 1.6% , autumn 3.1% winter 26.8% and spring 68.5% (43).

 Kargar *et al.* collected 138 stool sample from inpatient children less than 5 at Shahid Dastgheib and Namazi Hospital in Shiraz, Iran, with acute diarrhea from December 2006 to November 2007. 34.78% of samples were reported as group A rotavirus. 70.83% of infections occurred in children under 2 years old with the highest incidence (20.83%) in children between 9 to 11 months of age and the lowest incidence occurred in children 0 to 2 months and 48 to 60 months. The highest incidence of rotavirus (45.83%) was seen in autumn and the lowest (8.33%) in spring (44).

Emamghorashi *et al.* in 2008 reported the rate of 67.6% for rotavirus infection in inpatient children between 2-60 months of age with acute diarrhea (2).

Farahtaj *et al.* detected 24.6% rotavirus from children 6-60 months of age with diarrhea (inpatient and outpatient at Markaz Tebbei Koudakan in Tehran, Iran) from November 2003 to October 2004. In this study the percentage of the most common genotype was 59.2% for G1P[8], SGII and the rate of other genotypes were 15.5% for G9P[8] SGII ( which reported for the first time from in Iran in this study ), 2.8% for G1P(10) SGI and 1.4% for G12P[8] SGII (which are unusual genotypes ) 5.6% for the strains from reassortment among usual genotypes like G1P[4] SGII. In this study also mixed infections with G1+G4P[8] SGII and G1+G9P[8] SGII detected (45).

Kargar *et al.* detected 34.78% rotavirus in inpatient children (<5 years). In this study the percentage of the most common genotype was 27.08% for G4, and the rates of other genotypes were 6.25% and 2.08% for G1 and G2, respectively. Nontypeable and mixed infections were reported in 31.25% and 33.34% of inpatient children with diarrhea, respectively. In this study 70.83% of infections occurred in children younger than 24 months of age (46).

Zaraei Mahmoodabadi *et al.* in 2006 reported the prevalence of rotavirus infection in children less than 5 in Tehran 35% inpatient children and 20.88% outpatient children (47). 

Savadkoohi *et al.* in 2007 conducted a survey in children with gastroenteritis in North of Iran to determine the role of rotavirus, adenovirus and astrovirus. They reported that rotavirus was detected in 61.1% of patient; astrovirus and adenovirus were detected in 2.4% and 2.9% of patients, respectively. The highest incidence of rotavirus was occurred 29.8% in newborn (6 to 12 months). The lowest isolation rate of rotavirus was in spring (8.33%) and the highest was in autumn (45.83%) (48).

Sadeghian *et al.* found rotavirus prevalence in children (<6 years, 2006-2007) from Mashhad (a city in NorthEast Iran). Rotavirus was detected in 28.8% of patient. The highest prevalence of rotavirus was observed in winter season (44.4%) and in cases younger than 24 months age (69%) (3, 48).

Maleki *et al.* studied on the incidence of rotavirus in patients (<3 years) suffering from acute diarrhea in Tehran. They reported rotavirus infection was detected in 24.6% of patient with acute diarrhea (49).

Kargar *et al.* detected 28.37% rotavirus from children 1-60 months of age with diarrhea (inpatient at Motahary Hospital, Marvdasht, Iran) From January 2007 to December 2008. In this study the percentage of the most common genotype was for G types 60.0% for nontypeable 12.50% for G2 12.5 % for G4 10.05 % and for G1 5% . The highest prevalence of rotavirus was in children less than 24 months of age (69%). The highest incidence of rotavirus infection was in summer (52.5%) and the lowest in winter (7.5%) and 72.91% of rotavirus infections were occurred in children less than 2 years of age (50).

Khoshdel *et al.* conducted a study in children 6-60 months of age who were admitted in hospital due to diseases except diarrhea during December 2010 and October 2011 in Shahrekord, Iran. In this study the incidence of rotavirus related infection was 30% and the prevalence of genotypes was G1 (20%) , G9 (20%) , mixed genotypes: G1+G9 (13.3%), G1+G4 (6.7%), and G1+G8 (3.3%) , G1+G3 (3.3%), respectively (51).

Rahbarimanesh and Modaress *et al.* during Oct 2004 to Sep 2008 determined the prevalence of rotavirus in children between 1 to 60 months of age in two pediatric hospitals in Tehran, Iran 19% .The most common genotypes were G1 (76.3%) G4 (11.5%), G8 (0.8%), P [4] (9.2%) and P[8] (66.4%) and the prevalence of mixed genotypes in this study were G1P[8] (53.4%), followed by G1P[4] (9.2%) and G4P[8] (4.6%). In this survey 3.1% of total samples were mixed types and G1G2/-P (1.5%), G1G4P[4] (0.8%) and G1G4P[8] (0.8%) respectively. 91.6% of total samples were G types and 77.1% P types and 8.4% of total samples were detected as P[8] type and 22% of samples were only G type (45,52).

Ghorashi *et al.* reported the rate of rotavirus infection in hospitalized children <36 months of age with acute diarrhea in Tabriz from October 2007 to October 2009, 55.6%. The most of rotavirus infections were occurred in winter and autumn (53).

Kargar* et al.* detected 46.2% rotavirus from children less than 5 with diarrhea in Jahrom, Iran. In this study 46.2% of samples were positive for rotavirus and the percentage of the most common genotype was 30.66 % for G4 and the rate of other genotypes were 17.33 % for G1,13.34 % for G2, 2.67% for G3 and 2.67% for G9 and 2.67% for mixed genotypes. The highest rate of rotavirus related infection (22.69%) was in winter and the lowest was in summer (4.29%) (54).

Hassanzadeh *et al.* determined the incidence of 28.8% in rotavirus infection in children between 3-12 months of age with diarrhea in Shiraz, Iran. The highest prevalence of rotavirus was in children less than 24 months of age (69%). The highest incidence of rotavirus infection was in children between 7 to 9 months of age in November and May (55).

In 2012 Moghim *et al.* reported rotavirus was positive in 12.66% of children with gastroenteritis (< 3 years) in Isfahan ,Iran (56).

Jadali *et al. *conducted a survey to estimate the incidence of rotavirus related acute diarrhea in children (<5 years) in Bandar Abbas, Mashhad, Shiraz, Tabriz and Tehran (five main cities of Iran) from April 2010 to March 2011. Of total samples 28.8% were positive for rotavirus, 16.77% in Bandar Abbas, 14.42% in Shiraz, 8.97% in Tehran, 7.76% in Mashhad and 7.56% in Tabriz. The highest incidence of rotavirus gastroenteritis was occurred in winter (41.26%) (57).

Kajbaf* et al.* carried out an epidemiological survey in Ahwaz (a city in southwest of Iran) in children between 1-60 months of age during September to August 2009-2010 who were referred to medical care centers with acute diarrhea. Rotavirus infection was detected in 35% of patients. The seasonal distribution of rotavirus diarrhea was 38.1% in autumn, 25.4% in winter, 19% in spring and 17.5% in summer (58).

Sharifi-Rad *et al.* performed a study in children less than 1 to 12 months during the winter of 2013. They showed that the incidence of rotavirus was 70.20% (59).

Kargar *et al*. conducted a study during September 2010 to August 2011 in hospitalized children <5 years in Yasuj, Iran. The incidence of rotavirus study was 28.26%. The result of this study showed G8 was predominant genotype with 46.16% frequency and the other genotypes were nontypeable genotype 40.39%, G2 7.69%, G4 1.92%, G1 1.92% and mixed genotypes 1.92%. The highest incidence of rotavirus gastroenteritis was occurred autumn (48.08%), and the lowest rate of infection happened in spring (5.77%) (60).

**Rotavirus vaccine**

Vaccines are an efficient and accessible proceeding for safeguarding from rotavirus complications and also for prevention of rotavirus diarrhea. According to WHO estimation, globally 23% of susceptible newborns had rotavirus vaccine in 2015 with coverage approximately fourfold lower than the estimated overall coverage for polio and diphtheria-tetanus-pertussis vaccines (86%) (20, 61, 62). From May 2016, a group of countries has entered rotavirus vaccine into their internal immunization policies. Presently, 3 kinds of vaccine including live, oral, attenuated (rotavirus strains of human and/or animal origin that can replicate in the human intestinal epithelial cells) are available for Rotavirus (62). Generally, there is two oral live attenuated rotavirus vaccines that are presented globally including the monovalent (RV1) and the pentavalent (RV5) vaccines (20). The RV1 (lyophilized and liquid) also known as RV1 is an oral vaccine that originated from a G1P (8) strain that was isolated from a case of infantile gastroenteritis. Another type of oral vaccines for Rotavirus is the RV5 which includes five reassortant rotaviruses developed from human and bovine derived rotavirus strains. Many challenges remain until we assess to the global effective rotavirus vaccines that can prevent severe and fatal cases of rotavirus infection in children. These second-generation rotavirus vaccines, RotaTeq (RV5) and RotarixTM (RV1), have been permitted in over 100 countries. Despite the high efficacy determined by the vaccines in studies in developed countries, the WHO Strategic Advisory Group of Experts (SAGE) on immunization, postponed making a recommendation for international practice in 2006, awaiting the accessibility of vaccine data survey from developing countries, including some African and Asian countries. Other additional pieces of evidence have found on live oral vaccines effectiveness variations between various populations, with efficacy being lower in developing country populations with the maximum rate of disease. Although both vaccines were confirmed to be effective in preventing severe rotavirus disease due to homotypic and heterotypic rotavirus vaccine strains, long-term monitoring is needed to assess possible selection pressure. Additionally, as both available vaccines are developed from only a limited number of strains of rotavirus, there have been considerations that they would not adequately protect from serious infection induced by non-vaccine rotavirus strains (20, 62, 63).

Antirotavirus vaccination (Rotarix) in Brazil started since March 2006. Carvalho-Costa *et al.* studied the incidence of rotavirus during the vaccination program before and after universal vaccination from January 2005 to December 2009, the prevalence of rotavirus was 33.8% in 2005, 23.7% in 2006, 16.8% in 2007, 22.9% in 2008, and 18.3% in 2009. The prevalence of genotype G1P[8] was respectively 14%, 12.3% , 9.5% , 0.7% , 20.4% in 2005 , 2006 , 2007 , 2008 , 2009 and the rates of detection G2P[4]/ G2P[NT] were 9% in 2005, 49% in 2006, 66% in 2007, 85% in 2008, and 37.5% in 2009 , G3P[8]/G3P[NT] was detected in 8.7% in 2005, 3.5% in 2006, and 5.7% in 2009; G9P[8]/G9P[NT] was detected in 52% in 2005, 22% in 2006, 12.3% in 2007, 3.2% in 2008, and 3.4% in 2009 (64).

Rotarix antirotavirus vaccine in Belgium introduced to the market in 2006 and RotaTeq in 2007. The average rate of rotavirus infection was 19.0% during 1986 to 2006. The percentage of rotavirus positive cases were 12.4%, 9.6% and 6.4% in 2007, 2008, and 2009 respectively. G3 and G4 together were responsible for more than 80% of the rotavirus gastroenteritis cases during 2003–2004 caused by G3 and G4 genotypes and 10% of cases were caused by G1 and G9 genotypes. G9 was responsible for 46.2% of rotavirus gastroenteritis and G1 genotype was responsible for 23.7%. During 2005–2006 G1 genotypes with the rates of 70.3% were predominant cause of rotavirus gastroenteritis which followed by G9. During 2006 to 2007 after the introduction of Rotarix antirotavirus vaccine 31.5% of rotavirus gastroenteritis caused by and 28.3% of rotavirus gastroenteritis caused by G1 and G9 together.

From 2007 to 2008 G1 and G2 genotypes were respectively responsible for 44.4% and 36.5% of rotavirus gastroenteritis and G12 reemerged after they were first reported in 2003–2004 in Belgium. During 2008–2009 the prevalence of G2 genotype was the highest (38.5%), G3 and G9 each was responsible for 20.5% of rotavirus gastroenteritis and G1 was 15.4%. From 2007 to 2009 P[8] had the highest incidence of P-type followed by P[4] (61.8% and 59% for P[8] 2009 and 38.1% and 41.1% for P[4] during 2007-2008 and 2008- respectively) (65).

In Japan, monovalent rotavirus (RV) vaccines introduced in 2011 and pentavalent rotavirus (RV) vaccines introduced in 2012. The average rate of roavirus gastroenteritis hospitalization before vaccination (2007-2011) for children less than 5 years of age was 4.2 cases per 1000 person-years.

The rate of rotavirus gastroenteritis hospitalization after vaccination years (2011–2012, 2012–2013, 2013–2014 and 2014–2015) for children less than 5 years of age were 3.0, 3.5, 0.8 and 0.6 cases per 1000 person-years, respectively.

During 2007 to 2011, the highest predominant rotavirus genotypes were G3P[8] (61.5–75.0%) and G1P[8] (11.1–28.2%) and between 2011 to 2012 and 2012 to 2013, the highest predominant rotavirus genotypes were G1P[8] (78.1–96.9%). During 2013 to 2014, all of the detected specimens was G2P[4] and from 2014 to 2015, the highest genotype was G1P[8] (66.7%) 

From 2010 to 2011, the highest rotavirus genotype was G3P[8] (48.5%), and the second genotype was G1P[8] (39.4%). During 2011 to 2012 and 2012 to 2013, the highest rotavirus genotypes were G1P[8] 73.9% and 91.3%, respectively. From 2013 to 2014, the highest genotype was G2P[4] 83.3% and between 2014 to 2015, all detected specimens was G1P (8) (66).

Rotarix is recently licensed for newborn and children in Yemen and was disclosed in 2012. The prevalence of hospitalization due to rotavirus diarrhea declined from 43.79% in 2009 to 10.54% in 2014.

The highest dominant genotypes from January 2009 to July 2012 ( before vaccination ) were G2P [4] (55.0%), and the second dominant genotype was G1P [8] (15.0%) while from January 2013 to December 2014 (after vaccination) G1P[8] (31%) was the highest dominant genotype, and the second dominant genotype was G9P [8] (27.5%) (67).

Afrad *et al.* performed a study in rural Matlab, Bangladesh from June 2006 to May 2012 to determine changing profile of rotavirus genotype. From 2006 to 2012, 20.3% of samples were rotavirus positive with the highest incidence 24.5% in 2008 to 2009 and the lowest 17.3% in 2011 to 2012.

Rotarix (monovalent rotavirus vaccines) and Rotateq (pentavalent rotavirus vaccines) introduced in Matlab during 2007–2012 and showed less than 45% vaccine efficacy. During 2006 to 2007 the prevalence of rotavirus was 18.6% and G9P[8] was the predominant (34.8%), followed by G1P[8] and G2P[4] (each of them 13%), the rates of other genotypes were 8.7% for G9P[6], 4.3% for each of G4P[8] and G1P[6], 21.7% for mixed genotypes G1G9P[8] (13%), G1G4P[6] (4.3%), G2G9P[8] (4.3%) (Figure 1).

During 2007 to 2008 the prevalence of rotavirus was 19.3% and G2P[4] was the predominant (35.1%), followed by G1P [8] (27%) the rates of other genotypes were 24.3% for G9P[8], 2.7% for each of G2P[6], G2P[8] and G12P[6], 5.4% for mixed genotypes G2G9P[8] (2.7%), G1G2G9P[8] (2.7%).

During 2008 to 2009 the prevalence of rotavirus was 24.5% and G1P[8] was the predominant (35.1 %), followed by G9P[8] (23.8%) the rates of other genotypes were 9.5% for G2P[4], 7.1% for G12P[6], 4.8% for G1P[6], 2.4% for each G12P[8], G2P[8], G9P[6] and 4.8% for mixed genotypes G1G9P[8] (4.8%).

During 2009 to 2010 the prevalence of rotavirus was 20.5% and mixed genotypes were the predominant (37.5%), G1G2P[4]:6.3%, G2G9P[4]:3.1%, G2G9P[8]:6.3%, G1G2G9P[8]:3.1%, G1G2G9P[4] P[6] P[8]:3.1, G1G2G9P[6] P[8]:6.3, G1G2G9P[6]: 3.1%, G2G9P[4]P[8]: 3.1%, G1P[6] P[8]: 3.1, followed by G9P[8] (25%) the rates of other genotypes were G2P[4] (12.5%) , G12P[6] (9.4%) , G1P[8] and G12P[8] each one (6.3%), G9P[6] (3.1).

From 2010 to 2011 the prevalence of rotavirus was 20.9%. G12P[8] was the predominant (48.1%) followed by G1P[8] (18.5%). the rates of other genotypes were 11.1% for G9P[8], 3.7 for G2P[4] and G12P[6] (each of them) and 14.8 for mixed G/P G1G12P[8] (14.8%).

From 2011 to 2012 the prevalence of rotavirus was 17.3%. G9P[4] was the predominant (31.8% followed by G2P[4] (27.3%) the rates of other genotypes were13.6% for G1P[8], 13.6 for G12P[8] ,4.5% for G12P[4] and 4.5 for mixed G/P (G2G9G12P[8] (14.8%) and 4.5% of positive cases were G/P untypeable. From 2008 to 2009 the dominant genotype was G1P[8] with the rate of 42.9%, this rate decreased to 6.3% in 2009–2010. From 2006 to 2007 G9P[8] genotype was the predominant genotype (34.8) and gently reduced while G2P[4], which was rare before 2004, became dominant in 2005 to 2008 and 2011 to 2012. G12P[8] genotype was a trace genotype before 2009 and increased to 48.1% in 2010 to 2011 (68).

A recent survey on children with primary immunodeficiency diseases (PID) revealed rotavirus as an important infectious agent in Iran. Parvaneh L *et al.* studied 38 PID patients including severe combined immunodeficiency (SCID), common variable immunodeficiency (CVID), X-linked agammaglobulinemia (XLA), and hyper-IgM (HIgM) syndrome with chronic diarrhea and found different infectious agents such as Rotavirus group A, Giardia lamblia, salmonella spp and Candida albicans as the most important viral, parasitic, bacterial and fungal agents (69). So, it is really important to access to diagnostic kits and investigate rotavirus in these susceptible patients. 

The Rotarix vaccine s was introduced in the National Immunization Program of Brazil in March of 2006, the prevalence of rotavirus was 11.12% from 2002 to 2006 (pre-vaccine period) and 5.07% from 2007 to 2011 (post-vaccine period). During 2002 to 2005 G1P[6] (18.2%) was the highest frequently diagnosed genotype and in 2006 G2P[4] in 2006 (33.3%) and from 2007 to 2011 (15.2 %). During 2002 to 2005 in terms of G types 50% of rotavirus positive cases were G1, 25% were G9 and 25% were not identified and in terms of P types. 50% were not identified and 25% were P[8] and 21% were P(6) and 4% were P (4).

 In 2006 in terms of G types, 68% of rotavirus positive cases were G2, 4% were G9 and 28% were not identified and in terms of P types 56% were P (4), 8% were P (8), 8% were P (6) and 50% were not identified.

During 2007 to 2011 in terms of G types 59% of rotavirus positive cases were not identified and 29 % were G2, 12% were G1 and in terms of P types 59% were not identified , 35% were P[4] and 6% were P[8] (70).

Since December 2010 RotaTeq has introduced into the Israeli. The average of rotavirus hospitalization infection was 56% during 2008–2010 (pre-vaccination) and 22% during 2012–2015 (post vaccination period). During 2008–2010 (pre-vaccination) the most frequently genotype was G1P[8] (35.3%), followed by G2P[4] (15.5%). The rate of mixed genotypes, G3P[8], G4P[8], G9P[8] genotypes were 19.5%, 8.8%, 4.3%, 4.3% respectively. During 2010–2015 (post-vaccination) the most frequently genotype was G1P[8] (48.6%) followed by G3P[8] (21.5%) The rate of G9P[8], G12P[8], G9P[8] genotypes were 15.9% and 4.7% mixed genotypes (71).In 2008, 78% of rotavirus genotypes were G2P[4, 6] and G2P[6]. During 2009 the most frequently genotypes were G1P[6] (32%), G1+G9P[6] (13%), and G9P[6] (8%). After the introduction of rotavirus vaccine G2P[4] emerged and became dominant in 2010 and 2011 with the rate of 54% , 86% respectively while during 2012 , G2P[4] decreased to 32% and the rate of other genotypes G9P[8], G9P[4], G2P[8], and G9P[6] were 20% , 14% , 7% ,and 5% respectively. G2P[4], was dominant in 2008 (47%). In 2010 , 2011 , and 2012 the rate of G2P[4] genotype was 54%, 86%, and 32% respectively (72). Rotavirus vaccine (Rotarix and RotaTeq) was introduced to Venezuela since 2006. 

Before introducing rotavirus vaccine in Venezuela, the dominant genotypes were G1P[8], G3P[8] and G4P[8] and mild rate of G2P[4] and slight rate of G9P[8] and G8P[14]. After vaccination, 22.6% diagnosed with immunochromatography assay which 82% of positive cases were confirmed by PAGE assay and therefore the final prevalence of rotavirus was 18.5%. The positive cases were characterized for the G (VP7) and P (VP4) and showed 46.7% G2P[4] genotype, 38.2% G1P[8] genotype, 2.4% G8P[14] genotype, 1.2% G9P[8] genotype , 1.2% G1P[4] genotype and 0.6% for each of the following combination G3P[8], G2P[6], G4P[4] and G8P[4] , 3.6% samples were mixed genotype (G1-G2/P[8]-P[4]) and 4.2% of samples were untypeable (73). 

In Iran, several epidemiological studies indicated the importance of RV infection in infants and children. Accordingly, national RV vaccination program is highly recommended (Figure 2). 

## Conclusion

 Rotavirus is one of the most important infectious agents that cause the mortality of infants and children less than 5 years old worldwide. After approval, wide application and introduction of rotavirus vaccine into national immunization programs in developed countries, the prevalence of RV infection and mortality rate have been decreased significantly. However, it is necessary to continue nationwide surveys to control and monitor any cases of RT gastroenteritis to elucidate the remaining.

## Conflict of interests

The authors declare that they have no conflict of interest.
